# Neurodegeneration in Friedreich's Ataxia: From Defective Frataxin to Oxidative Stress

**DOI:** 10.1155/2013/487534

**Published:** 2013-07-09

**Authors:** Cláudio M. Gomes, Renata Santos

**Affiliations:** ^1^Instituto Tecnologia Química e Biológica, Universidade Nova de Lisboa, Avenida da República, 2784-505 Oeiras, Portugal; ^2^Development of the Nervous System, IBENS, Ecole Normale Supérieure, 46 rue d'Ulm, 75230 Paris Cedex 05, France

## Abstract

Friedreich's ataxia is the most common inherited autosomal recessive ataxia and is characterized by progressive degeneration of the peripheral and central nervous systems and cardiomyopathy. This disease is caused by the silencing of the *FXN* gene and reduced levels of the encoded protein, frataxin. Frataxin is a mitochondrial protein that functions primarily in iron-sulfur cluster synthesis. This small protein with an **α**/**β** sandwich fold undergoes complex processing and imports into the mitochondria, generating isoforms with distinct N-terminal lengths which may underlie different functionalities, also in respect to oligomerization. Missense mutations in the *FXN* coding region, which compromise protein folding, stability, and function, are found in 4% of FRDA heterozygous patients and are useful to understand how loss of functional frataxin impacts on FRDA physiopathology. In cells, frataxin deficiency leads to pleiotropic phenotypes, including deregulation of iron homeostasis and increased oxidative stress. Increasing amount of data suggest that oxidative stress contributes to neurodegeneration in Friedreich's ataxia.

## 1. Friedreich's Ataxia: Origin, Clinical Features, and Neurodegeneration

The main feature of Friedreich's ataxia (FRDA) disease is progressive and unremitting ataxia [1–3]. The first symptoms appear at puberty but onset of the disease can occur from infancy to after 60 years [4]. Major neurologic signs include gait and limb ataxia, tendon areflexia, dysarthria, sensory loss, and pyramidal signs [5]. Cardiomyopathy is a frequent symptom and is associated with a severe prognosis, particularly in young patients [6]. Some patients can also develop skeletal deformation, ocular abnormalities, hearing loss, and diabetes [5]. The neuropathology of FRDA involves degeneration of the dorsal root ganglia, peripheral nerves, the spinal cord, and the dentate nucleus in the cerebellum [7]. Patient nerves show axonal neuropathy with loss of large myelinated fibers and an increase in the number of small unmyelinated fibers [8, 9].

FRDA is caused by a GAA trinucleotide repeat expansion in the first intron of the *FXN* gene [10]. The majority of patients are homozygous for the trinucleotide expansion but in 4% of patients, one allele presents point mutations in the coding region. Expanded alleles lead to the inhibition of *FXN* expression resulting in decreased levels of the encoded protein, frataxin [10, 11]. The transcriptional repression of the *FXN* gene induced by the GAA expansion is due to arrest of RNA polymerase II progression and to heterochromatin-mediated gene silencing [12–16]. Frataxin is a mitochondrial protein involved in cellular iron use and maintenance of the redox status [4]. Although the function of frataxin has been a matter of debate since its discovery, it is now generally accepted that its primary function is in iron-sulfur cluster biosynthesis [17–20].

Frataxin is expressed in all cells of eukaryotic organisms. However, the levels of *FXN* mRNA and frataxin show tissue specificity that partially correlates with the sites of disease. In humans, the highest levels of expression are found in the heart and spinal cord and lower levels are observed in the cerebellum, liver, skeletal muscle, and pancreas [10]. The differential sensitivity of the tissues to frataxin deficiency is not clear but may depend on the cellular metabolism and/or on the somatic instability of expanded GAA triplet repeats [4, 21]. For dorsal root ganglia, one of the first tissues affected in FRDA patients, it was shown that somatic instability starts during embryonic development and continues throughout life, resulting in progressive, age-dependent accumulation of larger GAA triplet repeat expansions [21, 22]. Nevertheless, understanding why only certain tissues are sensitive to frataxin depletion will contribute to a better understanding of the pathophysiology of the disease. Oxidative stress has been suggested to be one of the major inducers of neurodegeneration, but the underlying mechanisms are not fully understood [4, 23, 24]. Antioxidant therapy has been tested since the discovery of the gene with different molecules with limited success in stopping the progression of the disease; however, it is a potential target to treat the disease, and new molecules are being tested.

## 2. The Frataxin Protein: Structure and Function

Human frataxin (FXN) is a small protein, which is involved in the mitochondrial biogenesis of iron-sulfur clusters (ISCs). These inorganic structures are essential redox cofactors found in several respiratory and metabolic enzymes within mitochondria. Although frataxin function remains to be fully elucidated at the molecular level, a wealth of biochemical data and the determination of three-dimensional structures from several homologues of FXN, particularly yeast frataxin (Yfh1), have contributed to substantial progress on understanding frataxin function [25–29]. 

**Figure 1:**
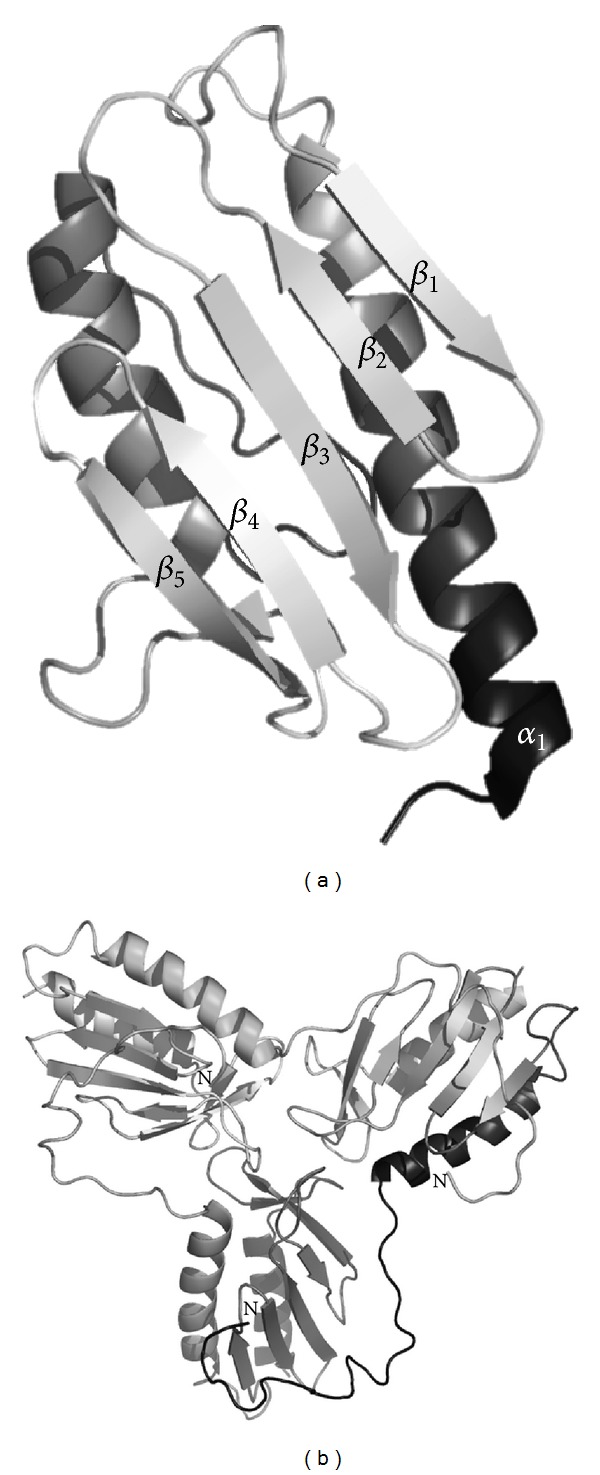
Structures of the frataxin monomer and trimer, denoting the typical *α*/*β* fold in which the *α*-helices pack against the *β*-sheet strands. (a) Structure of the human frataxin monomer (PDB: 3S4M). (b) Structure of the yeast frataxin trimer (PDB: 3OEQ). Note that in the trimer structure, unwinding of the N-terminal *α*-helix affords an interaction with a *β*-sheet from the nearby subunit.

The frataxin fold is characterized by a planar *α*-*β* sandwich motif, comprising two terminal *α*-helices, alongside with five antiparallel *β*-strands that make up two planes which are intersected by additional *β*-strands, thus composing the structure (Figure 1(a)). Several frataxins have been shown to undergo oligomerization reactions, assembling into trimers, hexamers, and larger order 24- and 48-mer assemblies, although there is no consensus in the community regarding the possibility of these reactions taking place *in vivo* or on their functional relevance (Figure 1(b)). Frataxin function involves participating in larger molecular assemblies with the several components from the mitochondrial ISC assembly machinery, and the molecular details of these interactions are only now starting to be elucidated with the disclosure of possible interactions taking place in such quaternary complexes [30–32]. Nevertheless, other still controversial roles have been proposed for frataxin, including delivery of iron to ferrochelatase for heme synthesis, recovery of the oxidatively damaged [3Fe4S] cluster in aconitase, and iron storage (reviewed in [17]). 

One important region in the frataxin fold is the so-called acidic region, which comprises a set of acidic residues located within the first *α*-helix and the edge of the first *β*-strand (*α*1 and *β*1 in Figure 1(a)), that are involved in low affinity iron-binding [33]. Nevertheless, iron-binding is essential for the interaction between Yfh1 frataxin and the Isu scaffold protein from the ISC machinery [34]. Studies on Yfh1 focusing on a set of functional mutants in the acidic region have shown that charge-to-neutral alterations in the ridge do not abolish iron-binding, but rather decrease binding affinity [35]. Indeed, frataxin iron-binding capacity is quite robust, as even upon changing five of the most conserved residues from the putative iron-binding region, at least two iron atoms per monomer can be bound. This study has also elicited an interesting trade-off between frataxin function and stability of the fold. Although these negative charges have a functional role, at the same time, they significantly impair Yfh1 stability as their replacement results in a dramatic increase of protein stability while reducing conformational plasticity. The acidic ridge has thus evolved to favor function over stability, therefore highlighting its importance on frataxin structure. On the other hand, mutations of conserved residues of the portion of the acid ridge found in the *β*-sheet, affected interaction with the scaffold protein Isu, but did not affect overall protein stability [35].

Frataxin processing and cleavage upon import into the mitochondria results, in some cases, in the production of diverse isoforms with distinct structural properties, especially in what concerns oligomerization propensity [36–38]. Human frataxin is a 210-amino acid protein which upon expression in the cytosol is imported into the mitochondria matrix, where it undergoes a complex processing resulting in isoforms with different lengths at the N-terminus [36–38]. Identified isoforms include shorter frataxin versions (processed at positions 42- and 56-) and longer ones (processed at positions 78- and 81-). In humans, the most abundant products seem to be the monomeric form FXN(81-201) which predominates in fibroblasts, alongside with the oligomerization prone longer FXN(42-120) isoform, which is abundant in the heart, cerebellum, and dividing fibroblasts [39]. In fact, distinct N-terminal processing of human frataxin has been suggested to result in proteins with distinct functionalities in what concerns interactions with the ISC machinery proteins and iron-binding properties [17, 39]. In yeast, one major form of processed frataxin is detected in the mitochondria (corresponding to Yfh1 52-174), which seems to be present mostly in the form of trimers, unless protein overexpression takes place which then generates larger oligomers [40]. Stress and increased iron uptake seem also to induce the formation of higher order Yfh1 oligomers [41].

One aspect which remains to be fully investigated is whether frataxin may be itself a target for oxidative modifications in the context of FRDA in which very low frataxin levels are present with iron accumulation in an environment prone to oxidative reactions. To establish a proof of principle, the susceptibility of frataxin to amino acid carbonylation and nitration was investigated [42]. The results on FXN(91-210) showed that residues in the *β*-sheet surface (Tyr143, Tyr174, Tyr205, and Trp155) were preferential targets, and that modifications did not alter substantially iron binding or protein stability. Interestingly, the strictly conserved Trp155, which is mutated in early onset patients, is a hot spot for modifications leading to the speculation that this could be an effective mechanism to modulate frataxin interactions and thus its function [42].

## 3. FRDA-Related Frataxin Mutations Result in a Loss of Function Misfolding Disease

**Figure 2:**
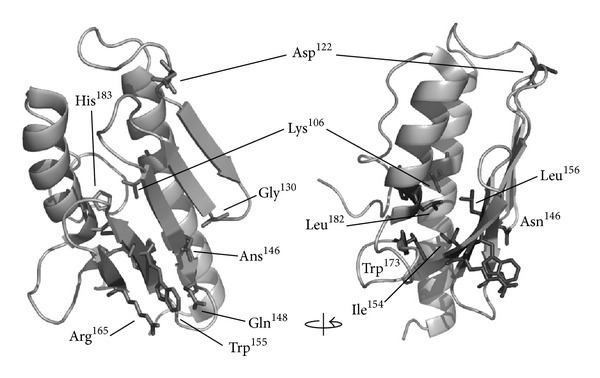
Mapping of amino acids mutated in FRDA compound heterozygous patients. FRDA mutations indicated on the human frataxin structure (PDB: 3S4M): Lys106Ser, Asp122Tyr, Gly130Val, Asn146Lys, Gln148Arg, Ile154Phe, Trp155Arg, Leu156Pro, Arg165Cys, Trp173Gly, Leu182Phe, Leu182His, and His183Arg. The arrow denotes a 90° rotation.

A small fraction of FRDA patients (around 4%) are compound heterozygotes for the intronic GAA trinucleotide repeat expansion, carrying in the other allele a missense mutation in the coding region of the *FXN* gene (Figure 2). These mutations are useful to understand how loss of functional frataxin impacts on FRDA physiopathology. Some of these mutations can be grouped according to FRDA symptoms severity: for example, whereas the FXN-p.Ile154Phe and FXN-p.Trp155Arg mutations lead to severe FRDA, the mutations FXN-p.Gly130Val and the FXN-p.Asp122Tyr account for milder clinical symptoms, although the latter has a very low prevalence [43]. Overall, the link between a point mutation in frataxin and the disease physiopathology remains unclear. Whereas first approaches shed light onto possible molecular mechanisms of disease by analyzing effects of mutations on protein folding and stability [44, 45], more recently mutations have also been studied in the context of cell models [46] and distinct frataxin isoforms with different N-terminal processing [47], and for some mutants, crystal structure information has been obtained [48].

The initial studies on FRDA frataxin mutants focused on the aggressive phenotype FXN-p.Ile154Phe and FXN-p.Trp155Arg variants, studied in the background of the shorter processed FXN(91-210) protein [44]. The former is among the most common clinical mutation, and affects an isoleucine residue at the protein core, and thus is expected to directly affect the structure and stability of the protein fold. The latter affects a conserved tryptophan residue at the protein surface in the exposed region of a *β*-sheet. The study has shown that these FRDA frataxin variants retained the native fold under physiological conditions but were thermodynamically destabilized in respect to normal frataxin had a higher tendency towards proteolytic degradation, and iron-binding was only partly impaired, nevertheless with concomitant protein aggregation [44]. The implication of these findings was that the severe mutations did not abolish the expression of the FRDA frataxin variants, suggesting that these proteins are formed under physiological conditions. Interestingly, a FRDA murine fibroblast cell model based on the FXN-p.Ile154Phe has corroborated this observation *in vivo*, as transgenic expression of this pathological mutation partly rescued endogenous frataxin deficiency [46]. Further, these studies also evidenced decreased activity of iron-sulfur proteins and accumulation of iron [46], in agreement with the poorer iron-binding ability which had been observed *in vitro* [44]. 

The evidence that the pathogenic mechanism in FRDA mutant frataxin results from frataxin misfolding and instability suggests that, although quite different from other neurodegenerative diseases involving toxic aggregation and metal ions [49], FRDA in compound heterozygous patients can be classified as a protein misfolding disease [50]. In order to further explore this possibility, a broader study encompassing also frataxin mutations yielding the milder FRDA forms FXN-p.Asp122Tyr and FXN-p.Gly130Val was undertaken [45]. The conclusion from those studies showed that FRDA frataxins exhibit distinct degrees of conformational defects that impair folding and function. For example, protein degradation propensity does not correlate necessarily with disease severity, as the two milder mutations were found for example to undergo proteolysis at higher rates than the severe ones, which rather seem to expose more particular part of the protein. An exploration of folding defects caused by mutations resulting in a change in the soluble : insoluble ratio upon recombinant expression showed that the severe FXN-p.Trp155Arg and FXN-p.Ile154Phe variants are mostly expressed as insoluble peptides, indicating that these mutations affect early folding [45]. Interestingly these results were corroborated by an investigation of the effects of these mutations in the context of the isoforms FXN(42-210) and FXN(81-210) in cell-free and different cellular models [47]. In addition, this study has shown that the FXN-p.Trp155Arg mutation destabilizes FXN(42-210) to a greater extent, as the mutation in this form makes the protein more susceptible to *in vivo* degradation than in the FXN(81-210) form. Likewise, the FXN-p.Ile154Phe variant in FXN(42-210) has a strongly compromised solubility; however, in FXN(81-210) yields a folded polypeptide when expressed in different cells types [47], in agreement with the results obtained *in vitro* for the purified recombinant mutant variant [45].

## 4. Oxidative Stress in Frataxin-Deficient Cells and Neurodegeneration

Oxidative stress is a central feature of FRDA disease and still a privileged target for therapy [4, 23, 24]. An increasing amount of data from different organisms support the hypothesis that frataxin-deficiency causes a deregulation in the antioxidant defenses, which result in oxidative stress and pathology [4, 24, 51, 52]. Increased levels of prooxidant molecules such as H_2_O_2_ and superoxide have been detected in yeast, *Drosophila* and FRDA patient cells [40, 53–58]. In addition, frataxin deficiency increases the cellular sensitivity to a wide variety of prooxidants in yeast [58, 59], *Caenorhabditis elegans* [60], *Drosophila* [56, 61, 62], mouse [63], and patient FRDA cells [64–68]. 

The eukaryotic cellular response to oxidative stress involves the induction of detoxifying enzymes such as superoxide dismutases (SODs), an increase in glutathione and NADPH synthesis, a decrease in the reduced to oxidized glutathione ratio and glutathionylation of target proteins [69]. SODs convert superoxide into H_2_O_2_ and are upregulated upon oxidant insult. However, in fibroblasts from FRDA patients, unlike those from healthy controls, SODs are not upregulated in response to low doses of H_2_O_2_, oligomycin, and iron [57, 65, 66]. In yeast, the anaerobiosis to aerobiosis transition is an inducer of oxidative stress in Δ*yfh1* cells as a result of transcriptional repression of several genes encoding critical antioxidant enzymes (SODs, catalases, glutaredoxins, and thioredoxins) and of decrease of total glutathione levels [58, 70]. Glutathione is a major antioxidant molecule in eukaryotic cells. Furthermore, reversible protein S-glutathionylation is a post translational modification that provides protection of protein cysteines from irreversible oxidation and also functions in the transduction of redox signals [71]. Several studies show that frataxin deficiency leads to the impairment of glutathione homeostasis [52, 53, 72–75]. A significant increase in the glutathione pool bound to proteins was observed in blood samples, fibroblasts, and lymphoblasts from FRDA patients and yeast Δ*yfh1* cells [72, 74, 75]. In addition, Pastore et al. found that in patient fibroblasts actin was glutathionylated, which caused disassembly of the filaments [74]. The analysis of autopsy samples from the spinal cord of FRDA patients also showed abnormal microfilament polymerization [76].

The discovery of actin glutathionylation and the demonstration that the Nrf2-dependent Phase II antioxidant pathway is defective in patient fibroblasts provided the first mechanism for the reduction of antioxidant defenses in human frataxin-deficient cells [57]. Nuclear factor erythroid 2-related factor 2 (Nrf2) is a key transcription factor that responds to oxidants by inducing expression of antioxidant enzymes to restore redox homeostasis in the cell. The Nrf2 activity is regulated by the actin-associated Keap1 (Kelch-like ECH-associated protein 1), which is an adaptor protein for the Cul3-dependent E3 ubiquitin ligase complex. Under normal conditions, Keap1 sequesters Nrf2 in the cytoplasm and promotes its rapid degradation via ubiquitination [77]. Under oxidative stress conditions, the cysteines of the Keap1 protein became oxidized, and the activity of the Cul3-Keap1 ubiquitin E3 ligase complex is reduced. As a consequence, Nrf2 is stabilized and translocated to the nucleus where it binds to DNA sequences of the *cis*-acting antioxidant responsive element (ARE), activating the expression of genes encoding antioxidant enzymes like SODs, catalase, glutathione S-transferase, and NADH quinone oxidoreductase [77]. In fibroblasts from FRDA patients, Keap1 is not associated with actin, and Nrf2 is distributed diffusely in the cell [57]. In addition, in FRDA fibroblasts treated with oligomycin or tert-butylhydroquinone, Nrf2 fails to translocate to the nucleus and the antioxidant Phase II genes are not induced [57]. This phenotype can be reversed by 24 h treatment with the catalase mimetic Euk134; the actin stress fibers were reorganized, and Nrf2-signalling was restored, highlighting the role of H_2_O_2_ in FRDA pathophysiology. Recently, it was shown that the *PIP5K1B* gene that is located upstream of the *FXN* gene is also silenced in FRDA patient lymphocytes and fibroblasts [48]. The pip5k1*β* protein is a key regulatory factor of actin cytoskeleton dynamics, and its down-regulation in fibroblasts causes actin network destabilization [48]. Therefore, it is very likely that in FRDA patient cells, *PIP5K1B* gene silencing contributes to actin disassembly, but a direct evidence of actin glutathionylation in cells deficient for pip5k1*β* protein is missing.

Microarray analysis of dorsal root ganglia from YG8R frataxin-deficient mice and controls revealed significant differences in genes belonging to the thiol antioxidant, the myelination, and the axon transport functional categories [52]. In addition, the authors found not only a decreased expression of antioxidant genes that are regulated by Nrf2, but also Nrf2 (at mRNA and protein levels) in YG8R dorsal root ganglia compared to controls. However, their observations using different cell lines (HeLa, fibroblast, and ND7/23 dorsal root ganglion neuron cell lines and patient lymphoblasts) did not support deficient translocation of Nrf2 to the nucleus upon oxidant insult; Nrf2 is being mainly localized in the nucleus in frataxin-deficient cells. Consistent with these data, D'Oria et al. found no increase in Nrf2 expression in frataxin-silenced NSC34 cells (derived from the fusion of neuroblastoma cells and spinal cord motor neurons) compared to controls upon treatment with oxidized glutathione neither an increase in the Nrf2 nuclear fraction [78]. Neurons and fibroblasts are completely different cells types, and it is possible that in neurons antioxidant defense regulation depends primarily on Nrf2 levels while in fibroblasts it is the actin-driven translocation to the nucleus the major regulator. 

Chronic inflammation and activity of glial cells are important factors that lead to neurodegeneration in other diseases, such as Alzheimer's disease and amyotrophic lateral sclerosis [79]. In FRDA, degeneration affects different types of neurons in the peripheral and central nervous systems, and it is likely that other cell types are also affected by frataxin deficiency. Several reports suggest that Schwann cells (SCs) could be specifically affected and that peripheral neuron loss would be a secondary event [9, 80]. Schwann cells are glial cells specialized in the myelination of axons in the peripheral nervous system. Signaling between neurons and SC plays an essential role in SC proliferation, survival, migration, and myelination [81]. On the opposite, SC also contributes to the preservation of axon integrity [82]. Analysis of the dorsal roots in patient autopsy samples shows differences in the myelination of thin fibers and reduced number of SC [9]. In addition, patient sural nerve autopsies also suggest participation of SC in neurodegeneration since although the large myelinated fibers are significantly reduced the total number of axons per unit area is similar to that of controls [83]. In agreement with these observations, *in vitro* studies using human SC lines showed frataxin knock-down by siRNA blocks cell cycle progression at G_2_M, and this is followed by an inflammatory response and an increase in cell death [80]. Treatment of these cells with antiinflammatory and antiapoptotic drugs rescued the death phenotype. Altogether, these results suggest that defects in SC could be at the origin of hypomyelination or demyelination and axon degeneration in FRDA patients. 

## 5. Mitochondrial and Nuclear DNA Damage

Oxidative DNA damage is a natural consequence of aerobic metabolism that is exacerbated when cells are in oxidative stress conditions. ROS induce more than 20 lesions in the DNA, such as oxidized bases, apurinic/apyrimidinic (AP) sites, base deamination products, oxidized sugar fragments, and DNA strand breaks [84]. Damaged DNA bases, if not repaired, may have miscoding properties leading to mutation upon replication or by blocking progression of the replication fork [85, 86]. The most studied DNA lesion is the 8-oxo-7,8-dihydro-2′-deoxyguanosine (8-oxodG), a product of oxidation of guanine. Conflicting results have been obtained for the levels of urinary 8-oxodG between FRDA patients and controls, since a difference has not always been found [87, 88]. However, mitochondrial and nuclear DNA damage have been reported in human, mouse, and yeast frataxin-deficient cells [51, 89–92].

Mitochondrial DNA (mtDNA) loss was one of the first phenotypes reported for the Δ*yfh1* yeast cells [93–95] and for FRDA patient cells [96]. Karthikeyan et al. showed that strong frataxin depletion in yeast cells resulted in complete loss of mtDNA after 15 generations and that reduced frataxin levels resulted in only partial loss of mtDNA after 22 generations [91]. These results indicate that mtDNA loss is dependent on the frataxin levels in the cell. Recently, it was shown that in Δ*yfh1* yeast cells, mtDNA loss is oxygen exposition-dependent, with complete retention in anaerobic conditions [51]. Mitochondrial DNA lesions and significant loss in the heart, cerebellum, and dorsal root ganglia have been observed in FRDA patients [92, 96–98]. 

Decades ago, two reports showed evidence of nuclear DNA damage in skin fibroblasts and blood lymphocytes of FRDA patients in response to ionizing radiation and mutagens, respectively [99, 100]. These studies suggested that in these patients, an increased susceptibility to DNA damage and/or defective DNA repair pathways were present. In agreement with this hypothesis, the transcriptome profiling of total blood from 28 children with FRDA revealed the molecular signature of cell response to DNA damage [92]. Using quantitative PCR, the same authors showed increased number of mitochondrial and nuclear DNA lesions in blood cells from FRDA patients compared to controls. A link between frataxin expression, DNA-repair, and tumor initiation was observed in murine liver [89]. In addition, this work showed that overexpression of human frataxin in hamster fibroblasts was associated with a decrease in the nuclear mutation frequency.

Detailed studies on nuclear DNA damage and repair pathways have been performed in yeast. Analysis of diploid frataxin-deficient yeast cells showed evidence of chromosomal instability with higher levels of illegitimate mating, higher rate of spontaneous mutation, and increased sensitivity to the DNA-alkylating methyl methanesulfonate and to the replication inhibitor hydroxyurea than controls [90]. Furthermore, deletion of the glutathione peroxidase encoding gene *GPX1* in frataxin-deficient cells resulted in a marked increase in the nuclear mutation rate. These results led the authors to suggest that the increased spontaneous nuclear damage in Δ*yfh1* cells was caused by H_2_O_2_ generated in the mitochondria [90]. Consistent with this hypothesis, the analysis of the exposure of anaerobically grown Δ*yfh1* cells to oxygen showed that frataxin-deficiency in yeast cells leads to increased nuclear DNA damage [51]. The effect of oxygen was very rapid, 15 min after Δ*yfh1* cell exposition to oxygen, antioxidant levels, were decreased and DNA strand breaks were visible. At 30 min, cell cycle was arrested at G1/S, and mutation frequency was increased in Δ*yfh1* cells. Two hours later, the cells adapted to oxygen since the cell cycle was reinitiated but were in a chronic oxidative stress state with a high spontaneous mutation rate. The nuclear DNA lesions detected in Δ*yfh1* cells were primarily caused by oxidized bases and single-strand breaks, and the Apn1 AP-endonuclease of the base excision repair pathway was essential for the repair these DNA lesions [51]. Altogether, these observations suggest that DNA damage and repair could be important features in FRDA disease progression.

## 6. Antioxidant Therapy 

Different strategies for the discovery of effective treatments for FRDA disease are currently being developed or tested in clinical trials (see http://www.curefa.org/pipeline.html). Several groups have done significant advances in *FXN* gene replacement or frataxin replacement therapies [101, 102]. Other strategies target the expression of the GAA-expanded gene (e.g., the HDAC inhibitor RG2833 is in Phase I clinical trial) or the stabilization of the frataxin protein (e.g., erythropoietin is in Phase II clinical trial). In addition, molecules targeting physiological functions that are defective in patient cells, such as increase in mitochondrial functions and iron-sulfur cluster synthesis or decrease in oxidative stress and iron toxicity, are being developed or in clinical trials. The conclusion of a recent review of the results of all randomized controlled clinical trials with minimal duration of 12 months was that none of the pharmacological drugs tested, including idebenone, had a significant beneficial effect on the neurological symptoms in FRDA patients [103]. Nevertheless, several ongoing clinical trials are testing promising antioxidant molecules. The *α*-tocopheryl quinone EPI-A0001 is a potent antioxidant molecule that has been tested in a double-blind, randomized, placebocontrolled trial of two doses in 31 adults with FRDA for a short period (four weeks) [104]. Glucose tolerance was tested, and no statistical difference was observed in the Disposition Index, which is a measure of diabetic tendency. However, after four weeks of treatment a dose-dependent improvement in the Friedreich Ataxia Rating Scale score was observed indicating neurologic function improvement in the patients. The EPI-743 is a new drug based on vitamin E that modifies disease progression in patients suffering from inherited mitochondrial respiratory chain disorders [105]. A Phase II clinical trial is currently recruiting FRDA patients. OX1 (indole-3-propionic acid) is a naturally occurring drug compound that prevents oxidative stress by a combination of hydroxyl radical scavenging activity and metal chelation. This molecule is now in preclinical studies for toxicological safety.

## 7. Conclusion

In the last decade, major achievements have been obtained concerning the primary function of frataxin, and it is almost completely accepted that it is in iron-sulfur cluster synthesis. Also, the mechanisms that regulate *FXN* expression with expanded GAA repeats are well known. However, despite a strong international effort there is no proven therapy for FRDA that stops disease progression. One of the reasons could be that our knowledge of the physiology of frataxin-deficient cells, and in particular the neurons affected by the disease, is still reduced. A large number of studies show that frataxin-deficient cells present numerous pleiotropic secondary phenotypes, which arise from iron-sulfur clusters deficit, such as mitochondrial dysfunction, perturbed iron homeostasis, DNA damage and mutagenesis, and oxidative stress. However, other areas of research that are relevant for FRDA pathology and fundamental for the survival and functioning of neurons are still poorly studied; examples are mitochondria transport and dynamics, calcium homeostasis, nitric oxide signaling, and inflammation. A clearer picture of the effects of frataxin deficiency in neurons and other cell types that might participate in the degeneration process in FRDA disease is necessary.

The major problem until recently was the lack of good *in vitro* cellular and murine models. The mouse model that best recapitulates the disease features is the humanized mouse model (YAC transgenic mice containing the human *FXN* gene with 190 GAA repeats) [63]. However, in these mice the first phenotypes appear after 6 to 12 months of age, and there is a possibility of GAA trinucleotide repeat instability. New technologies that can allow the development of new and relevant models for FRDA disease research from stem cells have been developed. It is now possible to obtain individual types of neurons by patterning and differentiation of embryonic stem cells and induced pluripotent cells (iPS) or also by direct reprogramming of fibroblast or other cell types. A protocol for differentiation of peripheral nerve system neurons from human embryonic stem cells has been published [106]. The first reports of differentiation of neurons and cardiomyocytes from iPS cells from FRDA patients are encouraging, although the GAA trinucleotide repeats are unstable [107, 108]. Liu et al. differentiated sensory neurons and cardiomyocytes from iPS cells obtained from FRDA patients [107], and Hick et al. described mitochondrial deficits in the differentiated cells [109]. In the next years, the development of these new cell models will greatly contribute to our knowledge of the neurodegeneration process in FRDA disease.
